# (*E*)-2-[(2-Chloro­benzyl­idene)amino]­isoindoline-1,3-dione

**DOI:** 10.1107/S1600536811044898

**Published:** 2011-11-05

**Authors:** Hua-Jie Xu, Xue-Yue Jiang, Liang-Quan Sheng, Zhao-Di Liu

**Affiliations:** a Department of Chemistry, Fuyang Normal College, Fuyang Anhui 236041, People’s Republic of China

## Abstract

The title compound, C_15_H_9_ClN_2_O_2_, adopts an *E* configuration about the C=N double bond. The mean plane of the isoindoline ring system [maximum deviation = 0.011 (2) Å] is inclined to the chloro­benzene ring by 22.62 (8)°. In the crystal, mol­ecules are connected by C—H⋯O hydrogen bonds, forming chains that propagate along [010].

## Related literature

For background to and applications of amidrazones, see: Neilson *et al.* (1970 ▶); Lee *et al.* (1998 ▶); Radwan *et al.* (2007 ▶); Xu *et al.* (2009 ▶); Liu *et al.* (2011 ▶).
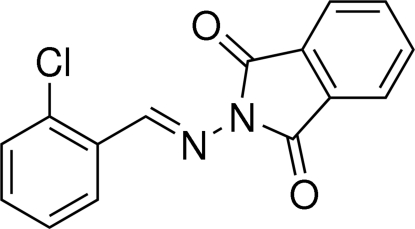


## Experimental

### 

#### Crystal data

C_15_H_9_ClN_2_O_2_
*M* *_r_* = 284.69
Monoclinic, 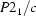

*a* = 12.991 (8) Å
*b* = 4.808 (3) Å
*c* = 23.757 (11) Å
β = 120.60 (2)°
*V* = 1277.2 (13) Å^3^
*Z* = 4
Mo *K*α radiation
μ = 0.30 mm^−1^
*T* = 293 K
0.20 × 0.20 × 0.10 mm

#### Data collection

Siemens SMART CCD area-detector diffractometer
Absorption correction: multi-scan (*SADABS*; Sheldrick, 1996 ▶) *T*_min_ = 0.942, *T*_max_ = 0.971
6727 measured reflections
2628 independent reflections
2017 reflections with *I* > 2σ(*I*)
*R*_int_ = 0.025

#### Refinement

*R*[*F* ^2^ > 2σ(*F* ^2^)] = 0.036
*wR*(*F* ^2^) = 0.104
*S* = 1.10
2628 reflections
181 parameters
H-atom parameters constrained
Δρ_max_ = 0.19 e Å^−3^
Δρ_min_ = −0.32 e Å^−3^

### 

Data collection: *SMART* (Siemens, 1996 ▶); cell refinement: *SAINT* (Siemens, 1996 ▶); data reduction: *SAINT*; program(s) used to solve structure: *SHELXS97* (Sheldrick, 2008 ▶); program(s) used to refine structure: *SHELXL97* (Sheldrick, 2008 ▶); molecular graphics: *SHELXTL* (Sheldrick, 2008 ▶); software used to prepare material for publication: *SHELXTL*.

## Supplementary Material

Crystal structure: contains datablock(s) global, I. DOI: 10.1107/S1600536811044898/su2334sup1.cif

Structure factors: contains datablock(s) I. DOI: 10.1107/S1600536811044898/su2334Isup2.hkl

Supplementary material file. DOI: 10.1107/S1600536811044898/su2334Isup3.cml

Additional supplementary materials:  crystallographic information; 3D view; checkCIF report

## Figures and Tables

**Table 1:** Hydrogen-bond geometry (Å, °)

*D*—H⋯*A*	*D*—H	H⋯*A*	*D*⋯*A*	*D*—H⋯*A*
C15—H15⋯O1^i^	0.93	2.52	3.421 (4)	163

Symmetry code: (i) 
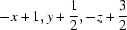
.

**Fig. 1.**
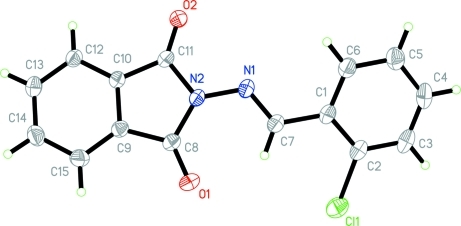
The molecular structure of the title compound, showing 30% probability displacement ellipsoids.

**Fig. 2.**
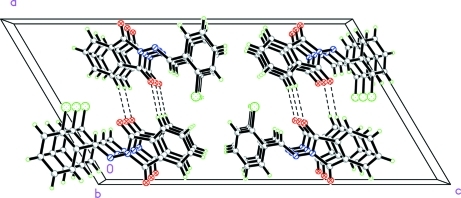
Crystal packing viewed along the b-axis. The intermolecular C-H···O hydrogen bonds are shown as dashed lines (details are given in Table 1).

## supplementary crystallographic information

### Comment

Amidrazones have been shown to act as important precursors or intermediates in the synthesis of various chemical compounds widely applied in industry (Neilson *et al.*, 1970), and the amidrazone moiety (C═NN) is an essential part of numerous molecules demonstrating high biological activity (Lee *et al.*, 1998; Radwan *et al.*, 2007). As part of an ongoing study of compounds based on the amidrazone moiety (Xu *et al.*, 2009; Liu *et al.*, 2011), we report herein on the crystal structure of the title compound, synthesized from 2-aminoisoindoline-1,3-dione and 2-chlorobenzaldehyde.

The molecular structure of the title compound is shown in Fig. 1. The C═N double bond adopts an E configuration. The isoindoline ring system [(N2,C8-C15); maximum deviation 0.011 (2) Å] makes a dihedral angle of 22.62 (8)° with the chorobenzene ring (C1-C6).

In the cystal, molecules are connected by C15-H15···O1 hydrogen bonds forming dimers, which stack along the b-axis direction (Fig. 2).

### Experimental

A solution of 2-aminoisoindoline-1,3-dione (0.16 g, 1 mmol) in 15 ml ethanol was added slowly to a solution containing 2-chlorobenzaldehyde (0.14 g,1 mmol) in 5 ml absolute ethanol, under heating and stirring, then the mixture was refluxed for 2 h. The mixture was then cooled to room temperature and the resulting solution left to stand in air for 15 days. Colourless prism-shaped crystals were formed on slow evaporation of the solvent.

### Refinement

All H-atoms were placed in calculated positions and treated as riding: C—H = 0.93 Å, with *U*_iso_(H) = 1.2*U*_eq_(parent C-atom).

### Crystal data

**Table:**

C_15_H_9_ClN_2_O_2_	*F*(000) = 584
*M**_r_* = 284.69	*D*_x_ = 1.481 Mg m^−^^3^
Monoclinic, *P*2_1_/*c*	Mo *K*α radiation, λ = 0.71073 Å
Hall symbol: -P 2ybc	Cell parameters from 2406 reflections
*a* = 12.991 (8) Å	θ = 3.1–27.1°
*b* = 4.808 (3) Å	µ = 0.30 mm^−^^1^
*c* = 23.757 (11) Å	*T* = 293 K
β = 120.60 (2)°	Prism, yellow
*V* = 1277.2 (13) Å^3^	0.20 × 0.20 × 0.10 mm
*Z* = 4	

### Data collection

**Table:**

Siemens SMART CCD area-detector diffractometer	2628 independent reflections
Radiation source: fine-focus sealed tube	2017 reflections with *I* > 2σ(*I*)
graphite	*R*_int_ = 0.025
φ and ω scans	θ_max_ = 26.5°, θ_min_ = 1.9°
Absorption correction: multi-scan (*SADABS*; Sheldrick, 1996)	*h* = −16→16
*T*_min_ = 0.942, *T*_max_ = 0.971	*k* = −6→5
6727 measured reflections	*l* = −29→23

### Refinement

**Table:**

Refinement on *F*^2^	Primary atom site location: structure-invariant direct methods
Least-squares matrix: full	Secondary atom site location: difference Fourier map
*R*[*F*^2^ > 2σ(*F*^2^)] = 0.036	Hydrogen site location: inferred from neighbouring sites
*wR*(*F*^2^) = 0.104	H-atom parameters constrained
*S* = 1.10	*w* = 1/[σ^2^(*F*_o_^2^) + (0.0444*P*)^2^ + 0.2726*P*] where *P* = (*F*_o_^2^ + 2*F*_c_^2^)/3
2628 reflections	(Δ/σ)_max_ = 0.001
181 parameters	Δρ_max_ = 0.19 e Å^−^^3^
0 restraints	Δρ_min_ = −0.32 e Å^−^^3^

### Special details

**Table:**

Geometry. Bond distances, angles etc. have been calculated using the rounded fractional coordinates. All su's are estimated from the variances of the (full) variance-covariance matrix. The cell esds are taken into account in the estimation of distances, angles and torsion angles
Refinement. Refinement of *F*^2^ against ALL reflections. The weighted *R*-factor *wR* and goodness of fit *S* are based on *F*^2^, conventional *R*-factors *R* are based on *F*, with *F* set to zero for negative *F*^2^. The threshold expression of *F*^2^ > σ(*F*^2^) is used only for calculating *R*-factors(gt) *etc*. and is not relevant to the choice of reflections for refinement. *R*-factors based on *F*^2^ are statistically about twice as large as those based on *F*, and *R*- factors based on ALL data will be even larger.

### Fractional atomic coordinates and isotropic or equivalent isotropic displacement parameters (Å^2^)

**Table:**

	*x*	*y*	*z*	*U*_iso_*/*U*_eq_	
Cl1	0.46943 (4)	0.33401 (14)	0.57225 (3)	0.0719 (2)	
O1	0.37871 (11)	0.9151 (3)	0.67721 (7)	0.0559 (5)	
O2	0.04114 (11)	0.5317 (3)	0.66062 (6)	0.0516 (4)	
N1	0.18301 (12)	0.4921 (3)	0.60576 (7)	0.0435 (5)	
N2	0.20315 (12)	0.6854 (3)	0.65344 (7)	0.0400 (5)	
C1	0.23630 (16)	0.2700 (4)	0.53596 (8)	0.0417 (6)	
C2	0.32867 (16)	0.1841 (4)	0.52658 (8)	0.0465 (6)	
C3	0.3118 (2)	−0.0195 (4)	0.48207 (10)	0.0578 (8)	
C4	0.2015 (2)	−0.1385 (4)	0.44524 (10)	0.0598 (8)	
C5	0.1073 (2)	−0.0545 (4)	0.45248 (9)	0.0577 (7)	
C6	0.12498 (17)	0.1482 (4)	0.49709 (9)	0.0494 (6)	
C7	0.25579 (16)	0.4778 (4)	0.58538 (9)	0.0463 (6)	
C8	0.29761 (14)	0.8758 (4)	0.68703 (8)	0.0400 (5)	
C9	0.27449 (14)	1.0096 (3)	0.73570 (8)	0.0382 (5)	
C10	0.17280 (14)	0.8941 (3)	0.73080 (8)	0.0371 (5)	
C11	0.12550 (15)	0.6817 (4)	0.67897 (8)	0.0386 (5)	
C12	0.13187 (16)	0.9757 (4)	0.77164 (8)	0.0427 (5)	
C13	0.19584 (17)	1.1782 (4)	0.81725 (9)	0.0496 (6)	
C14	0.29695 (17)	1.2934 (4)	0.82189 (9)	0.0536 (6)	
C15	0.33895 (16)	1.2110 (4)	0.78112 (9)	0.0484 (6)	
H3	0.37510	−0.07610	0.47700	0.0690*	
H4	0.18990	−0.27670	0.41520	0.0720*	
H5	0.03230	−0.13510	0.42720	0.0690*	
H6	0.06100	0.20540	0.50140	0.0590*	
H7	0.32090	0.59790	0.60160	0.0560*	
H12	0.06370	0.89710	0.76850	0.0510*	
H13	0.17040	1.23800	0.84540	0.0600*	
H14	0.33820	1.43010	0.85310	0.0640*	
H15	0.40740	1.28860	0.78440	0.0580*	

### Atomic displacement parameters (Å^2^)

**Table:**

	*U*^11^	*U*^22^	*U*^33^	*U*^12^	*U*^13^	*U*^23^
Cl1	0.0515 (3)	0.0882 (5)	0.0847 (4)	0.0060 (3)	0.0411 (3)	−0.0103 (3)
O1	0.0474 (7)	0.0648 (9)	0.0705 (9)	−0.0068 (6)	0.0410 (7)	−0.0087 (7)
O2	0.0510 (7)	0.0584 (8)	0.0576 (8)	−0.0154 (6)	0.0365 (6)	−0.0163 (6)
N1	0.0482 (8)	0.0460 (9)	0.0451 (8)	0.0008 (7)	0.0301 (7)	−0.0061 (7)
N2	0.0422 (8)	0.0420 (8)	0.0454 (8)	−0.0015 (6)	0.0293 (7)	−0.0056 (6)
C1	0.0516 (10)	0.0414 (10)	0.0415 (9)	0.0053 (8)	0.0305 (8)	0.0038 (8)
C2	0.0521 (10)	0.0507 (11)	0.0462 (10)	0.0107 (9)	0.0319 (9)	0.0065 (9)
C3	0.0763 (14)	0.0585 (13)	0.0582 (12)	0.0177 (11)	0.0485 (11)	0.0051 (10)
C4	0.0942 (16)	0.0474 (12)	0.0489 (11)	0.0047 (11)	0.0445 (12)	−0.0038 (9)
C5	0.0731 (13)	0.0559 (13)	0.0479 (11)	−0.0055 (11)	0.0335 (10)	−0.0033 (9)
C6	0.0555 (11)	0.0506 (11)	0.0502 (10)	0.0009 (9)	0.0329 (9)	−0.0018 (9)
C7	0.0483 (10)	0.0479 (11)	0.0527 (10)	−0.0014 (8)	0.0329 (9)	−0.0061 (9)
C8	0.0377 (8)	0.0412 (10)	0.0448 (9)	0.0044 (7)	0.0236 (8)	0.0034 (8)
C9	0.0358 (8)	0.0381 (9)	0.0418 (9)	0.0037 (7)	0.0205 (7)	0.0018 (7)
C10	0.0379 (8)	0.0367 (9)	0.0389 (8)	0.0025 (7)	0.0211 (7)	0.0001 (7)
C11	0.0394 (9)	0.0411 (10)	0.0423 (9)	0.0029 (8)	0.0258 (7)	0.0007 (8)
C12	0.0464 (9)	0.0444 (10)	0.0459 (9)	−0.0001 (8)	0.0297 (8)	−0.0024 (8)
C13	0.0588 (11)	0.0515 (11)	0.0453 (10)	0.0024 (9)	0.0314 (9)	−0.0060 (9)
C14	0.0556 (11)	0.0521 (12)	0.0464 (10)	−0.0036 (9)	0.0212 (9)	−0.0126 (9)
C15	0.0396 (9)	0.0494 (11)	0.0542 (11)	−0.0034 (8)	0.0224 (8)	−0.0031 (9)

### Geometric parameters (Å, °)

**Table:**

Cl1—C2	1.738 (2)	C9—C15	1.372 (3)
O1—C8	1.204 (3)	C10—C11	1.472 (3)
O2—C11	1.194 (3)	C10—C12	1.380 (3)
N1—N2	1.383 (2)	C12—C13	1.377 (3)
N1—C7	1.265 (3)	C13—C14	1.377 (3)
N2—C8	1.409 (3)	C14—C15	1.390 (3)
N2—C11	1.417 (3)	C3—H3	0.9300
C1—C2	1.390 (3)	C4—H4	0.9300
C1—C6	1.388 (3)	C5—H5	0.9300
C1—C7	1.462 (3)	C6—H6	0.9300
C2—C3	1.374 (3)	C7—H7	0.9300
C3—C4	1.367 (4)	C12—H12	0.9300
C4—C5	1.380 (4)	C13—H13	0.9300
C5—C6	1.370 (3)	C14—H14	0.9300
C8—C9	1.481 (3)	C15—H15	0.9300
C9—C10	1.382 (3)		
			
N2—N1—C7	118.91 (17)	O2—C11—C10	129.75 (19)
N1—N2—C8	130.37 (17)	N2—C11—C10	105.33 (16)
N1—N2—C11	117.57 (15)	C10—C12—C13	117.4 (2)
C8—N2—C11	111.74 (15)	C12—C13—C14	121.1 (2)
C2—C1—C6	117.38 (18)	C13—C14—C15	121.77 (18)
C2—C1—C7	121.40 (19)	C9—C15—C14	116.8 (2)
C6—C1—C7	121.2 (2)	C2—C3—H3	120.00
Cl1—C2—C1	119.77 (14)	C4—C3—H3	120.00
Cl1—C2—C3	118.74 (19)	C3—C4—H4	120.00
C1—C2—C3	121.5 (2)	C5—C4—H4	120.00
C2—C3—C4	119.7 (2)	C4—C5—H5	120.00
C3—C4—C5	120.3 (2)	C6—C5—H5	120.00
C4—C5—C6	119.6 (2)	C1—C6—H6	119.00
C1—C6—C5	121.5 (2)	C5—C6—H6	119.00
N1—C7—C1	119.14 (19)	N1—C7—H7	120.00
O1—C8—N2	126.02 (17)	C1—C7—H7	120.00
O1—C8—C9	128.90 (18)	C10—C12—H12	121.00
N2—C8—C9	105.08 (16)	C13—C12—H12	121.00
C8—C9—C10	108.94 (15)	C12—C13—H13	119.00
C8—C9—C15	129.43 (19)	C14—C13—H13	119.00
C10—C9—C15	121.62 (18)	C13—C14—H14	119.00
C9—C10—C11	108.86 (17)	C15—C14—H14	119.00
C9—C10—C12	121.37 (16)	C9—C15—H15	122.00
C11—C10—C12	129.75 (18)	C14—C15—H15	122.00
O2—C11—N2	124.92 (17)		
			
C7—N1—N2—C8	−2.3 (3)	C3—C4—C5—C6	−0.3 (3)
C7—N1—N2—C11	−175.13 (16)	C4—C5—C6—C1	−0.7 (3)
N2—N1—C7—C1	178.75 (15)	O1—C8—C9—C10	−178.23 (19)
N1—N2—C8—O1	4.2 (3)	O1—C8—C9—C15	0.4 (3)
N1—N2—C8—C9	−175.51 (16)	N2—C8—C9—C10	1.44 (19)
C11—N2—C8—O1	177.36 (18)	N2—C8—C9—C15	−179.98 (18)
C11—N2—C8—C9	−2.33 (19)	C8—C9—C10—C11	−0.09 (19)
N1—N2—C11—O2	−3.1 (3)	C8—C9—C10—C12	178.45 (16)
N1—N2—C11—C10	176.43 (14)	C15—C9—C10—C11	−178.80 (16)
C8—N2—C11—O2	−177.28 (18)	C15—C9—C10—C12	−0.3 (3)
C8—N2—C11—C10	2.29 (19)	C8—C9—C15—C14	−178.50 (17)
C6—C1—C2—Cl1	178.49 (14)	C10—C9—C15—C14	−0.1 (3)
C6—C1—C2—C3	−2.0 (3)	C9—C10—C11—O2	178.24 (19)
C7—C1—C2—Cl1	−2.3 (2)	C9—C10—C11—N2	−1.30 (19)
C7—C1—C2—C3	177.25 (18)	C12—C10—C11—O2	−0.1 (3)
C2—C1—C6—C5	1.8 (3)	C12—C10—C11—N2	−179.67 (17)
C7—C1—C6—C5	−177.41 (18)	C9—C10—C12—C13	0.4 (3)
C2—C1—C7—N1	−159.54 (18)	C11—C10—C12—C13	178.61 (18)
C6—C1—C7—N1	19.7 (3)	C10—C12—C13—C14	−0.2 (3)
Cl1—C2—C3—C4	−179.45 (16)	C12—C13—C14—C15	−0.1 (3)
C1—C2—C3—C4	1.0 (3)	C13—C14—C15—C9	0.3 (3)
C2—C3—C4—C5	0.2 (3)		

### Hydrogen-bond geometry (Å, °)

**Table:**

*D*—H···*A*	*D*—H	H···*A*	*D*···*A*	*D*—H···*A*
C15—H15···O1^i^	0.93	2.52	3.421 (4)	163

Symmetry codes: (i) −*x*+1, *y*+1/2, −*z*+3/2.

## References

[bb1] Lee, K., Hwang, S. Y., Hong, S., Hong, C. Y., Lee, C.-S., Shin, Y., Kim, S., Yun, S., Yoo, Y. J., Kang, M. & Oh, Y. S. (1998). *Bioorg. Med. Chem.* **6**, 869–876. 10.1016/s0968-0896(98)00044-3 9681152

[bb2] Liu, Z.-D., Xu, H.-J., Song, C.-F., Huang, D.-Q., Sheng, L.-Q. & Shi, R.-H. (2011). *Chem. Lett.* **40**, 75–77.

[bb3] Neilson, D. G., Heatlie, J. W. M. & Newlands, L. R. (1970). *Chem. Rev.* **70**, 151–170. 10.1021/cr60263a005 4904065

[bb4] Radwan, M. A. A. & El-Sherbiny, M. (2007). *Bioorg. Med. Chem.* **15**, 15, 2106–2119.

[bb5] Sheldrick, G. M. (1996). *SADABS* University of Göttingen, Germany.

[bb6] Sheldrick, G. M. (2008). *Acta Cryst.* A**64**, 112–122. 10.1107/S0108767307043930 18156677

[bb7] Siemens (1996). *SMART* and *SAINT* Siemens Analytical X-ray Instruments Inc., Madison, Wisconsin, USA.

[bb8] Xu, H.-J., Du, N.-N., Jiang, X.-Y., Sheng, L.-Q. & Tian, Y.-P. (2009). *Acta Cryst.* E**65**, o1047. 10.1107/S160053680901174X PMC2977729 21583865

